# Low-dose radiotherapy in patients with osteoarthritis: A multidisciplinary roadmap from biological rationale to definitive evaluation

**DOI:** 10.1016/j.ocarto.2026.100892

**Published:** 2026-11-03

**Authors:** Gilles Colin, Jean-François Kaux, Patrick Durez, François Lallemand, Pierre Blanchard, Clio Ribbens, Olivier Malaise, Jérémie Sellam

**Affiliations:** a Department of Radiation Oncology, Liege University Hospital, Belgium; b Department of Physical and Rehabilitation Medicine, Liege University Hospital, Belgium; c Rheumatology Department, Cliniques Universitaires Saint-Luc, Brussels, Belgium; d Pôle of Systemic and Inflammatory Rheumatic Pathologies, Institut de Recherche Expérimentale et Clinique, Université Catholique de Louvain, Brussels, Belgium; e Department of Radiation Oncology, Gustave Roussy Cancer Campus, Villejuif, France; f Centre for Research in Epidemiology and Population Health, INSERM U1018, Paris-Saclay University, Villejuif, France; g Rheumatology Department, Liege University Hospital, Belgium; h INSERM UMRS 938, Centre de Recherche Saint-Antoine, Sorbonne Université, France; i Rheumatology Department, Saint-Antoine Hospital, Assistance Publique-Hôpitaux de Paris, France

**Keywords:** Radiotherapy, Low-dose radiotherapy, Osteoarthritis, Inflammation, Benign condition, Anti-Inflammatory, Pain, Radiobiology

## Abstract

**Objective:**

To critically reassess low-dose radiotherapy (LDRT) in patients with osteoarthritis (OA) across the full translational pathway, from biological rationale to clinical efficacy and the requirements for definitive evaluation.

**Methods:**

This multidisciplinary narrative review appraised mechanistic, preclinical and clinical evidence for LDRT in OA, with particular emphasis on the translational relevance of experimental models and on randomized evidence, especially sham-controlled trials. Recent technical consensus, safety assessments and OA trial-methodology literature were also considered.

**Results:**

Preclinical studies support context-dependent immunomodulatory effects of LDRT involving endothelial–leukocyte interactions, immune-cell activation and joint-tissue crosstalk, but much of this evidence derives from reductionist or inflammatory models rather than chronic human OA. OA-specific experimental evidence remains limited. Clinically, two sham-controlled trials using 6 × 1 Gy were negative, whereas more recent studies using 6 × 0.5 Gy have reported positive signals that remain preliminary or methodologically limited. No responder phenotype has been validated. A definitive evaluation should therefore integrate clinically meaningful pain and/or functional impairment despite optimized standard OA care, prospective evaluation of inflammatory or imaging-based enrichment, biological target-engagement measures, standardized radiation delivery, rigorous sham-controlled OA outcomes, long-term safety, and assessment of clinical and health-system feasibility.

**Conclusion:**

There is a biological rationale for LDRT in OA, but current evidence remains insufficient to support routine use in patients with OA. Its future role should be defined through adequately powered, phenotype-informed sham-controlled trials conducted jointly by OA and radiation experts.

## 1 Introduction

Osteoarthritis (OA) is one of the most prevalent chronic musculoskeletal disorders worldwide and a major contributor to pain, disability, and health-care use [1], [2], [3], [4]. Despite this burden, no disease-modifying treatment is currently available. Current management remains largely symptomatic, and several pharmacological, surgical, and regenerative strategies have failed in rigorous randomized trials, reflecting limited preclinical translatability, substantial responses in placebo-controlled arms, and insufficient stratification of a heterogeneous disease [1], [2], [5], [6], [7], [8], [9], [10], [11], [12], [13], [14]. Education, exercise, weight management and multimodal rehabilitation therefore remain central to care, but important unmet needs persist.

Low-dose radiotherapy (LDRT, typically 6 × 0.5–1 Gy) has long been used for painful musculoskeletal disorders, particularly in Central Europe, and is now attracting renewed international interest [15], [16], [17], [18]. This expansion contrasts with persistent uncertainty regarding treatment-specific efficacy, mechanisms, patient selection and long-term safety, and with the absence of LDRT from major OA management guidelines [19], [20].

Recent OA-specific reviews, mechanistic syntheses, recommendations from the German Society for Radiation Oncology (DEGRO), and technical consensus documents illustrate both the momentum of the field and markedly different interpretations of the same evidence [16], [17], [21], [22], [23], [24], [25], [26]. Radiation-oncology-oriented publications have generally emphasized biological plausibility, accumulated clinical experience and implementation, whereas a recent OA-centered rheumatology perspective concluded that current evidence does not justify routine clinical use [26]. These contrasting perspectives highlight the need for an appraisal that integrates OA-specific methodological scrutiny with radiobiological and technical expertise.

Accordingly, this review examines the complete translational chain rather than providing another catalog of LDRT studies. We ask: (i) which proposed mechanisms are genuinely OA-specific rather than extrapolated from reductionist or acute inflammatory models; (ii) whether clinical evidence demonstrates benefit beyond sham when positive and negative findings are considered under comparable critical scrutiny; and (iii) which biological, phenotypic, technical and trial-design requirements should define a definitive OA evaluation. Our aim is neither to advocate implementation nor to dismiss LDRT on precedent, but to define the evidence required before it could credibly become part of clinical care for people with OA.

This multidisciplinary narrative review, bringing together four rheumatologists, three radiation oncologists, and one physical and rehabilitation medicine specialist, is based on a critical appraisal of the relevant literature, with particular emphasis on randomized controlled trials and OA-specific experimental evidence. The literature search was last updated on October 12, 2026.

## 2 From biological plausibility to OA-specific evidence

Recent reviews have extensively summarized the biological effects proposed for LDRT across OA and other musculoskeletal conditions [24], [27]. Here, we instead organize the evidence by translational proximity to chronic human OA, from reductionist inflammatory systems to patient-derived OA cells and OA-specific in vivo models. The strongest mechanistic literature remains non-OA-specific and suggests nonlinear, dose-dependent responses [27], [28]. Modulatory effects have repeatedly been reported around 0.3–0.7 Gy in several experimental systems, although effects have also been observed at higher doses depending on the model and endpoint. Heterogeneous models, endpoints and irradiation conditions preclude defining a universal or validated biological optimum (Fig. 1). Importantly, LDRT is not intrinsically anti-inflammatory [29], [30]. Its effects depend on dose, fractionation, timing, cell type, and inflammatory context. Supplementary Table 1 summarizes the preclinical evidence and its translational relevance.

**Fig. 1:**
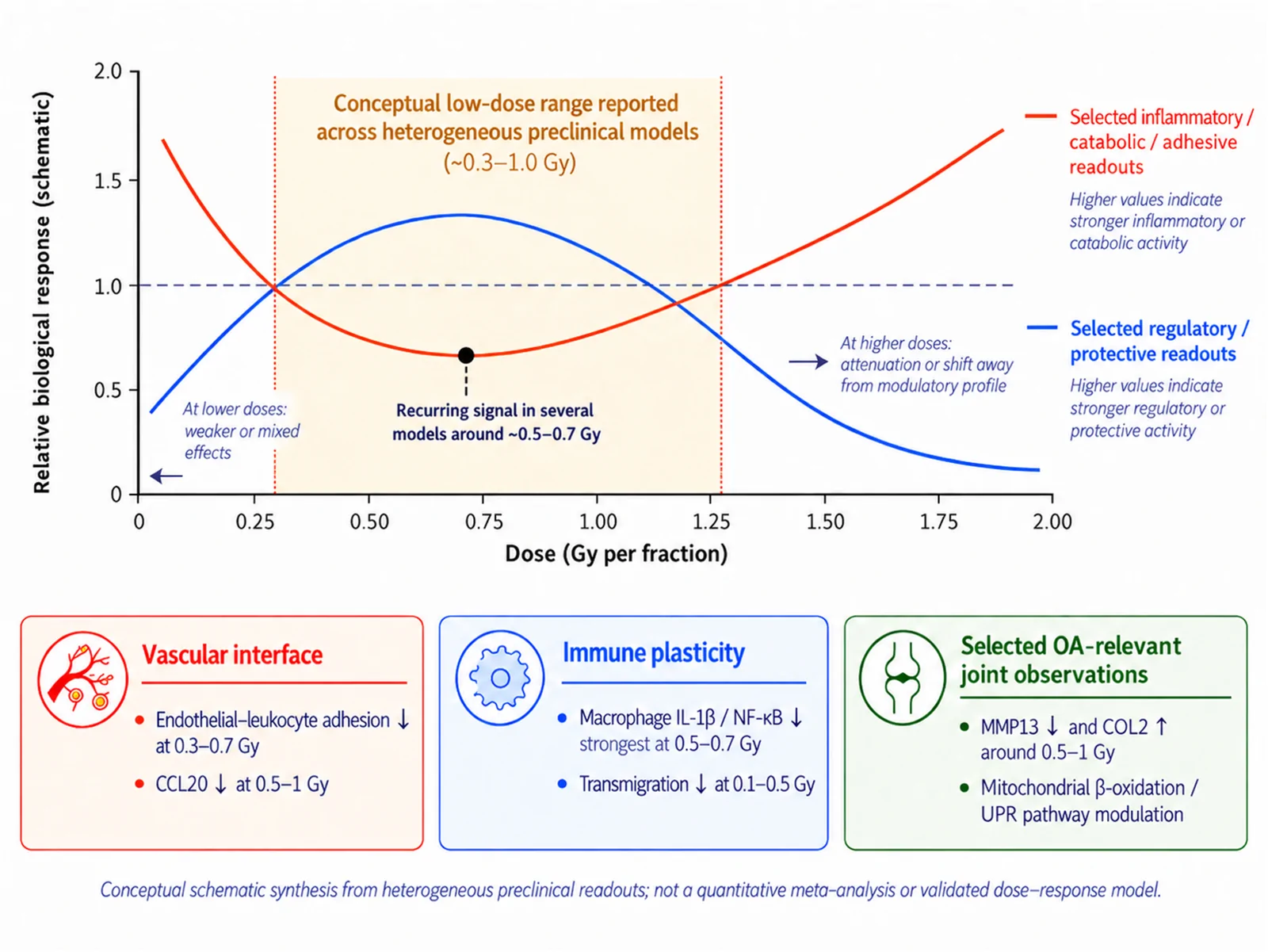
**Conceptual non-quantitative schematic of non-linear preclinical dose–response patterns reported in LDRT models relevant to osteoarthritis.** This figure summarizes recurring nonlinear dose–response patterns reported across preclinical low-dose radiotherapy (LDRT) studies relevant to osteoarthritis (OA). Most available data derive from reductionist inflammatory systems or non-OA models, with more limited evidence from OA-relevant human joint cells and OA animal models. Across endothelial, macrophage and joint-cell systems, LDRT has been associated with modulation of inflammatory, adhesive and catabolic pathways, including reduced endothelial–leukocyte adhesion, altered chemokine and cytokine signaling, modulation of macrophage activation, and reduced expression of selected catabolic mediators. Several studies have reported such effects within low-dose ranges around 0.3–1.0 Gy per fraction, but the position, magnitude and direction of the response vary substantially according to cell type, inflammatory context, irradiation timing, fractionation and biological endpoint. The figure is therefore intended as a conceptual synthesis of heterogeneous experimental observations and does not represent a fitted dose–response relationship or a validated therapeutic window. Rather than supporting a uniformly anti-inflammatory effect of “low-dose” irradiation, the available data suggest a context-dependent immunomodulatory intervention whose relevance to chronic human OA remains to be established. *The graphical development of this conceptual figure was assisted by ChatGPT (OpenAI), based on scientific content and instructions provided by the authors. The final figure was reviewed and validated by the authors*.

### 2.1 In vitro and ex vivo models: three OA-relevant biological axes

Mechanistic insights from in vitro and ex vivo systems converge on three main OA-relevant biological levels: vascular control of inflammatory cell entry, modulation of immune-cell activation states, and context-dependent responses of resident joint compartments.

#### 2.1.1 Vascular interface and leukocyte trafficking

A first mechanistic axis concerns the vascular interface. In inflammatory OA, the synovium can behave as an activated, vascularized tissue that supports leukocyte recruitment and sustains pain-relevant mediator networks [31], [32], [33], [34]. Across activated endothelial models, LDRT around 0.5 Gy reduces leukocyte or granulocyte adhesion and modulates inflammatory mediators, with evidence implicating transforming growth factor-β1 (TGF-β1) signaling, chemokine regulation and redox-dependent mechanisms [35], [36], [37], [38]. These effects are dose- and time-dependent, and in some models are restricted to selected low-dose ranges and early time points [39].

For OA, the translational implication is that LDRT may be most relevant where synovial inflammation and vascular activation are objectively present. However, these data are largely derived from reductionist or non-OA inflammatory systems, so they support target plausibility rather than proof of target engagement in human OA.

#### 2.1.2 Immune plasticity: myeloid modulation and selective lymphocyte radiosensitivity

A second axis involves immune-cell plasticity, particularly activated myeloid cells, and aligns with current OA concepts of whole-joint disease with an immune component [34]. Across macrophage models, LDRT can modulate inflammatory signaling and migratory behavior without uniformly suppressing cell viability or phagocytic function [40], [41], [42]. These effects are nonlinear and may vary according to host radiosensitivity [41].

The lymphoid compartment provides a complementary but less directly OA-specific signal. Human peripheral blood mononuclear cell (PBMC) studies show discontinuous apoptosis responses in the 0.3–0.7 Gy range, with variable sensitivity across lymphocyte subsets [43], [44]. In rheumatoid arthritis-derived samples, 0.5 Gy increased apoptosis of CD4^+^ T cells and CD19^+^ B cells, whereas fibroblast-like synoviocytes (FLS) required higher doses to show a clear apoptotic response [45].

These data should not be over-extrapolated to OA, but support preferential modulation of activated immune circuits rather than uniform immunomodulation.

#### 2.1.3 Joint compartments and tissue crosstalk

Because OA is a whole-joint disease, LDRT effects cannot be interpreted solely through immune-cell models. Resident joint cells may also contribute to the biological response.

In inflammatory arthritis-derived FLS, LDRT modestly reduces pro-inflammatory mediator production and alters macrophage responses to stromal cues, supporting the idea that irradiation can reshape synovial cell–immune cell crosstalk rather than act as a simple cell-intrinsic switch [46], [47].

OA-specific resident cell data are limited but important. In interleukin-1β-stimulated human OA chondrocytes and synoviocytes, LDRT reduced matrix metalloproteinase-13 expression and increased type II collagen protein within the 0.5–1 Gy range, without acute viability loss [48]. Transcriptomic analyses suggested nonlinear effects on mitochondrial and stress-response pathways [48], whereas senescence-related responses remain incompletely characterized [49].

The subchondral bone compartment may also be affected. Ex vivo osteoclastogenesis assays suggest that LDRT can reduce osteoclast resorptive activity [50], whereas cells derived from noninflamed joints show only limited basal responses [51].

Together, these observations suggest that LDRT effects may be amplified in activated or inflamed tissue environments, reinforcing the rationale for inflammatory or imaging-active OA phenotyping. Whether these compartment-specific signals translate into a coherent biological response when the full joint ecosystem is preserved remains a key question for dedicated in vivo OA models.

### 2.2 In vivo models (non-OA-specific)

Inflammatory arthritis models provide proof of principle that LDRT can modulate joint inflammation in vivo. Across adjuvant-induced, collagen-induced and K/BxN models, irradiation has reduced clinical inflammation and, in some experiments, structural damage through effects on inflammatory mediators, immune-cell populations and bone-remodeling pathways [45], [46], [52], [53], [54], [55]. Responses vary with disease phase, with no significant clinical effect reported when irradiation was delivered during the chronic phase of adjuvant arthritis [56].

These models therefore support biological plausibility but cannot establish efficacy in OA, where inflammation is generally lower-grade and embedded within a degenerative whole-joint process.

### 2.3 In vivo OA-specific models

OA-specific models are fewer but particularly important because they more directly address disease-relevant mechanisms. In a rat post-traumatic OA model induced by anterior cruciate ligament transection or destabilization of the medial meniscus, fractionated low-dose irradiation reduced cartilage histologic severity at 10 weeks, with Osteoarthritis Research Society International (OARSI) scores decreasing from 3.7 in nonirradiated animals to 2.8 after 5 × 0.5 Gy and 1.8 after 5 × 1 Gy, alongside reduced tartrate-resistant acid phosphatase (TRAP)-positive osteoclast activity [48]. In the monoiodoacetate model, a more inflammatory and pain-dominant system, knee irradiation reduced cartilage damage and synovitis, improved pain-related measures, and was associated with reduced joint inflammatory mediators, increased M2-like macrophage polarization and attenuation of spinal neuroimmune activation [57].

These models provide OA-relevant proof of principle but represent distinct experimental contexts with important translational limitations. Surgically induced models reproduce post-traumatic mechanical destabilization over a highly compressed timescale, whereas the monoiodoacetate model induces rapid chemically mediated cartilage injury and a prominent inflammatory pain response. Neither reproduces the slowly evolving, heterogeneous and age-associated biology of most human OA. Concordant effects across these models therefore strengthen biological plausibility, but cannot establish an optimal clinical dose range, mechanism of action, or responder phenotype in human OA.

### 2.4 Preclinical take-home messages and research gaps

Taken together, the preclinical evidence provides a biological rationale for LDRT in OA, although this rationale remains incomplete. Across the available models, the most recurrent signal is not direct cartilage repair, but context-dependent modulation of inflammation, including reduced leukocyte recruitment, altered macrophage activation, selective immune-cell radiosensitivity and modified synovial–cartilage–bone crosstalk. Preliminary OA-specific models also suggest possible effects on cartilage damage, synovitis, osteoclast activity, and pain-related neuroimmune pathways. However, evidence remains fragmented across heterogeneous models, doses and readouts. The key translational question is therefore not whether LDRT can modulate inflammation experimentally, but whether clinically deliverable schedules engage relevant targets in chronic human OA, and in which phenotypes. Importantly, biological findings from future human LDRT trials should in turn help determine which experimental models and mechanisms are genuinely relevant to the OA phenotypes being treated, rather than assuming direct translation from existing preclinical systems [58]. This bidirectional approach supports prospective biological phenotyping and enrichment strategies while avoiding assumptions of either broad efficacy or an established responder phenotype.

## 3 From historical use to treatment-specific clinical evidence

### 3.1 Historical context: a century-old treatment facing contemporary evidence standards

LDRT has been used for painful inflammatory and degenerative musculoskeletal disorders since the early twentieth century [59], [60]. Even early reports recognized that symptomatic improvement might reflect contextual effects, prompting controlled approaches including shielded sham irradiation [61]. Its use later declined in many countries as therapeutic alternatives expanded and late radiation risks became better understood, while persisting within more formalized practice frameworks in Germany and parts of Central and Eastern Europe [16], [59].

### 3.2 Does LDRT provide treatment-specific benefit in patients with OA?

The clinical literature on LDRT for OA is extensive but dominated by heterogeneous uncontrolled or retrospective studies [16], [62], [63], [64]. Because OA symptoms fluctuate and placebo responses (i.e., improvements observed in sham-treated patients, including placebo effects, natural symptom fluctuations, regression to the mean, and other nonspecific influences) can be substantial, treatment-specific efficacy can only be established reliably through appropriately blinded comparator trials, preferably using sham irradiation [65], [66], [67]. A recent meta-analysis reached broadly similar conclusions, although discrepancies in the reported study characteristics and potential overlap between trial populations in the pooled analysis warrant caution [68]. We therefore focused on the original sham-controlled evidence and interpret both positive and negative studies in light of their methodological strengths, limitations and clinical context. Table 1 summarizes the randomized evidence.

**Table 1:** Randomized clinical evidence and ongoing trials of low-dose radiotherapy for osteoarthritis.

Study	Design and population	Main eligibility criteria	Sample and baseline characteristics	LDRT regimen and comparator	Main outcomes	Interpretation
**Published randomized trials**						
Dutch knee OA trial[69], [70]	• Double-blind, randomized, sham-controlled trial • Knee osteoarthritis	• Age ≥50 years • ACR criteria for knee OA • NRS pain ≥5/10 • Inadequate response to analgesics and exercise therapy • Excluded: KL grade 4	• n = 27 (LDRT) vs 28 (sham) • Age: 62 vs 68 years • KL grade ≥2 in the index knee: 56% vs 61% • WOMAC pain/function: 59/60 vs 61/62 • NRS pain: 5.8 vs 5.4 • Symptom duration >5 years: 41% vs 48%	• 6 × 1 Gy (total 6 Gy) vs 6 × 0 Gy sham • Delivered over 2 weeks	• OMERACT-OARSI responders at 6 months: 41% vs 35% (NS) • At 12 months: 52% vs 44% (NS) • No treatment signal for objective inflammatory measures	• Pivotal negative sham-controlled trial in established symptomatic knee OA • A total dose of 6 Gy did not outperform sham in an unselected population with established symptoms
Dutch hand OA trial [70], [71]	• Double-blind, randomized, sham-controlled trial • Hand osteoarthritis	• Age ≥50 years • ACR criteria for hand OA • NRS pain ≥5/10 • Inadequate response to analgesics and exercise therapy • Excluded: Unilateral hand OA or KL grade 4 in >25% of hand joints	• n = 28 (LDRT) vs 28 (sham) • Age: 67 vs 63 years • Median number of joints with KL grade ≥2: 10.5 vs 6 • AUSCAN pain/function: 54/55 vs 56/59 • NRS pain: 6.1 vs 6.3 • Symptom duration >5 years: 68% vs 54% • Erosive hand OA: 50% vs 32%	• 6 × 1 Gy (total 6 Gy) vs 6 × 0 Gy sham • Delivered over 2 weeks	• OMERACT-OARSI responders at 6 months: 28% vs 41% (NS) • At 12 months: 31% vs 27% (NS) • No treatment signal for objective inflammatory measures	• Pivotal negative sham-controlled trial in established symptomatic hand OA • No benefit over sham in an unselected population with substantial radiographic burden
ArthroRad [72], [73]	• Multicenter, randomized, patient-blinded, active-dose comparator trial • Hand/finger and knee osteoarthritis	• Clinical OA of the knee and/or hand/fingers • Radiographic confirmation • Symptom duration >3 months	• 3-Gy arm: 77 hands and 33 knees • 0.3-Gy arm: 81 hands and 30 knees • Age: 68.2 vs 66.3 years • VAS pain: 59.3 vs 57.1 mm • Symptom duration: 56.2 vs 49.6 months • KOOS-PS: 20.5 vs 19.9 • SF-SACRAH: 21.3 vs 20.7	• 6 × 0.5 Gy (total 3 Gy) vs 6 × 0.05 Gy (total 0.3 Gy) • Twice weekly	• At 12 months, ≥30-point VAS improvement: 41% vs 44% • No consistent clinically meaningful between-dose difference in functional or quality-of-life outcomes	• Pain improved in both groups, but functional effects were limited, inconsistent across joint sites, and not dose-specific • The active-comparator design cannot establish treatment-specific efficacy
Makarova et al. [74], [75], [76], [77]	• Open-label randomized controlled trial • Knee osteoarthritis	• Knee OA according to Altman 1991 criteria • KL grades 0-2 • Baseline VAS pain ≥30 mm	• n = 146 (LDRT) vs 146 (control) • Age: 37.3 vs 39.8 years • Symptom duration: 9.7 vs 9.2 months • VAS pain: 57.1 vs 55.7 mm • KL grades 0/1/2: 10%/61%/29% vs 16%/59%/25%	• 10 × 0.45 Gy (total 4.5 Gy) plus NSAIDs/SYSADOA vs NSAIDs/SYSADOA alone • At least 48 h between fractions	• At 12 months, median VAS pain: 21.2 vs 54.9 mm • At 36 months, less MRI morphological progression (WORMS) with LDRT • At 10 years, disability: 9.5% vs 17.8% crude HR 0.49; adjusted HR 0.24	• Hypothesis-generating signal in early knee OA, but the study was open-label and lacked a sham comparator • Long-term disability and treatment-decision outcomes may be influenced by referral and decision bias • Interpretation is further limited by the limited international visibility of several follow-up publications and the absence of independent external confirmation
LoRD-KNeA [78]	• Multicenter, randomized, patient-blinded, sham-controlled trial • Knee osteoarthritis	• Age 50–85 years • KL grades 2-3 • Baseline walking pain 50-90	• n = 38 (3 Gy) vs 37 (0.3 Gy) vs 39 (sham) • Age: 67.3 vs 69.4 vs 67.6 years • VAS pain: 69.2 vs 68.1 vs 69.5 mm • KL grades 2/3: 16%/84% vs 30%/70% vs 36%/64%	• 6 × 0.5 Gy (total 3 Gy) vs 6 × 0.05 Gy (total 0.3 Gy) vs 6 × 0 Gy sham • Twice weekly	• At 4 months, OMERACT-OARSI responders: 70.3% with 3 Gy vs 41.7% with sham (p = 0.021) • Response with 0.3 Gy: 58.3%; not significantly different from sham	• Positive sham-controlled primary responder endpoint for the 3-Gy regimen; benefit persisted at 8 months but was not statistically significant at 12 months. • No consistent between-group differences were observed in continuous clinical, inflammatory, or MRI outcomes
Fazilat-Panah et al. [79]	• Double-blind, randomized, sham-controlled trial • Knee osteoarthritis	• Age ≥65 years • Knee OA confirmed by a rheumatologist using history, physical examination, and imaging	• n = 30 (LDRT) vs 30 (sham) • KL grades 1/2/3: 30%/67%/3% vs 13%/77%/10% • Symptom duration >6 months: 70% vs 57% • Baseline VAS categories 8/9/10 cm: 37%/63%/0% vs 27%/67%/7%	• 6 × 0.5 Gy (total 3 Gy) vs 6 × 0 Gy sham • Daily fractions	• Median VAS pain decreased from 9 to 6 cm at 6 months with LDRT and remained 9 cm with sham • Rank-based Mann-Whitney analyses showed between-group differences from months 1–6 for VAS pain and Lysholm function, with no baseline difference	• Positive sham-controlled symptomatic signal, but the trial was small, baseline pain was very high, and the median pain score remained unchanged in the sham arm. • Interpretation is limited by repeated rank-based monthly comparisons rather than prespecified longitudinal effect estimates
**Ongoing registered trials**						
**Radio-KO** NCT06887829	• Randomized, patient-blinded, sham-controlled trial • Knee osteoarthritis	• Age >50 years • KL grades 2-3 • ICOAP > A1 or B6	• Not yet reported	• 6 × 0.5 Gy (total 3 Gy) vs 6 × 0 Gy sham • Delivered over 2 weeks • Identical second course according to original allocation for non-responders	• Primary outcome: Change in the area under the curve of ICOAP scores at 6 months • Frequency of analgesic use	• Recruiting
**Open randomized hand OA trial** NCT07217405	• Open-label randomized controlled trial • Hand osteoarthritis	• Age >50 years • Mild, moderate, or severe radiographic hand OA (KL grades 2–4) documented within 24 months • VAS pain ≥40	• Not yet reported	• 6 × 0.5 Gy (total 3 Gy) vs corticosteroid injection • A second course is permitted for non-responders	• Change in VAS pain at 6, 9, and 12 months	• Recruiting
**IMMO-LDRT02** NCT05887284	• Double-blind, randomized, sham-controlled trial • OA of the hand, elbow, shoulder, knee, or foot	• OA diagnosed according to ACR criteria	• Not yet reported	• Initial course: 6 × 0.5 Gy (total 3 Gy) vs 6 × 0 Gy sham • A second course is permitted	• VAS pain assessed at multiple time points • Immunologic changes/target-engagement measures	• Recruiting

**Abbreviations:** ACR, American College of Rheumatology; AUC, area under the curve; AUSCAN, Australian/Canadian Hand Osteoarthritis Index; CSI, corticosteroid injection; HR, hazard ratio; ICOAP, Intermittent and Constant Osteoarthritis Pain; KL, Kellgren-Lawrence; KOOS-PS, Knee injury and Osteoarthritis Outcome Score Physical Function Short Form; LDRT, low-dose radiotherapy; NRS, numerical rating scale; NS, not significant; OA, osteoarthritis; OMERACT-OARSI, Outcome Measures in Rheumatology-Osteoarthritis Research Society International; RT, radiotherapy; SF-SACRAH, Short-Form Score for the Assessment and Quantification of Chronic Rheumatic Affections of the Hands; SYSADOA, symptomatic slow-acting drugs for osteoarthritis; VAS, visual analog scale; WOMAC, Western Ontario and McMaster Universities Osteoarthritis Index; WORMS, Whole-Organ Magnetic Resonance Imaging Score.

Ongoing trials were identified through a systematic ClinicalTrials.gov search performed on October 12, 2026. Eligible studies were randomized comparative interventional trials evaluating radiotherapy in OA, with at least one sham, control, alternative-dose, standard-care, or active-treatment comparator arm, and a status of Recruiting, Not yet recruiting, or Active, not recruiting.

### 3.3 Pivotal sham-controlled trials

The Dutch double-blind sham-controlled knee and hand OA trials remain pivotal because they tested LDRT against sham using accepted OA outcomes [69], [70], [71]. With 6 × 1 Gy, neither trial demonstrated superiority for Outcome Measures in Rheumatology–Osteoarthritis Research Society International (OMERACT–OARSI) response at 3, 6 or 12 months, nor a signal on objective inflammatory measures. These trials therefore provide sound evidence against routine use of this regimen in the populations studied. Participants nevertheless had established, highly symptomatic disease with substantial structural burden, often long duration with possible nociplastic pain components [80], a context in which a local anti-inflammatory intervention may be less effective [81]. Whether disease stage or inflammatory phenotype reduced treatment responsiveness remains a post hoc hypothesis, not an explanation for the negative findings, and requires prospective testing.

Placebo response remains central to interpretation [82]. A recent OA-centered rheumatology perspective similarly emphasized that symptomatic improvement in uncontrolled LDRT series cannot establish treatment-specific efficacy in a condition characterized by substantial placebo responses [26]. The Dutch trials showed no clinical advantage over sham irradiation, supporting the view that improvements reported in uncontrolled series may partly reflect placebo effects, natural symptom fluctuations, or regression to the mean. Importantly, sham-controlled radiotherapy trials are not intrinsically negative. In Ledderhose disease, the phase III LedRad trial demonstrated superiority of active radiotherapy over sham despite substantial improvement in both groups, illustrating the feasibility and value of sham-controlled radiotherapy designs [83].

### 3.4 Dose and fractionation: a central interpretative issue

Dose and fractionation remain central. Although some authors have argued that 6 × 0.5 Gy, with a predefined second course if required, best reflects contemporary German practice [84], [85], DEGRO recommendations include both 0.5 Gy and 1 Gy fractions [16]. Consequently, the key issue is not which regimen best reflects current practice, but whether any prospectively defined protocol demonstrates benefit beyond sham. If a second course is considered part of the intervention, it should be built into trial design with prespecified criteria and a mirrored sham pathway.

The ArthroRad trial illustrates the limits of active-comparator designs without sham control. This randomized, patient-blinded study compared 6 × 0.5 Gy with 6 × 0.05 Gy in painful knee or hand/finger OA [72], [73]. At 3 and 12 months, both groups improved, with no consistent clinically meaningful between-regimen advantage across pain, function, or quality-of-life outcomes [72], [73]. ArthroRad therefore demonstrates symptom improvement after an irradiation pathway, but does not establish treatment-specific efficacy or a clinically meaningful dose–response relationship.

### 3.5 Emerging positive trials

More recent sham-controlled evidence has reopened the field. In the patient-blinded LoRD-KNeA trial, 114 patients with knee OA were randomized to 6 × 0.5 Gy, 6 × 0.05 Gy, or sham irradiation [78], [86]. In the modified intention-to-treat analysis, the OMERACT–OARSI responder rate at 4 months was higher with 3 Gy than with sham (70.3% versus 41.7%; p = 0.021), whereas 0.3 Gy did not differ significantly from sham (58.3% versus 41.7%). The advantage with 3 Gy persisted at 8 months (76.5% versus 45.5%) but was no longer statistically significant at 12 months (66.7% versus 50.0%). Mean changes in Western Ontario and McMaster Universities Osteoarthritis index (WOMAC) subscales, Visual Analog Scale (VAS) pain, patient global assessment, inflammatory markers, rescue-medication use, and Magnetic Resonance Imaging (MRI)-based outcomes did not differ significantly among groups. No treatment-related toxicities were reported. These findings provide a positive sham-controlled signal on the prespecified responder endpoint, but its limited statistical durability and the absence of consistent differences in continuous clinical, biological, or imaging outcomes support the need for adequately powered confirmatory trials and prospective phenotyping.

Another double-blind, sham-controlled randomized trial in patients with knee OA reported a positive symptomatic signal with 6 × 0.5 Gy: median pain decreased from 9 to 6 over 6 months after LDRT while remaining at 9 under sham, alongside reduced analgesic use and improved performance status [79]. The sham-controlled design is an important strength. However, interpretation is limited by the small sample size (n = 60), unusually high baseline pain, absence of prospective inflammatory phenotyping, and an analysis based largely on repeated month-by-month rank comparisons rather than a clearly prespecified longitudinal treatment-effect estimate. An additional interpretative concern is the unchanged median pain score in the sham group, an unusual finding in OA pain trials given the typically substantial responses observed in sham groups [26]. This observation does not invalidate the trial but further argues for cautious interpretation and independent replication. The study therefore provides a clinically relevant positive signal, but should remain hypothesis-generating and does not, by itself, alter the current hierarchy of evidence.

An open-label randomized knee OA study and subsequent analyses reported improvements in pain and function, less MRI-defined progression, and lower long-term disability rates after orthovoltage radiotherapy added to conservative care [74], [75], [76], [77]. However, interpretation is limited by the absence of sham control and potential co-intervention and treatment-decision biases. More than 70% of participants had Kellgren-Lawrence (KL) grades 0–1 at baseline, with a mean age of approximately 38 years, raising questions about representativeness for established radiographic OA and the clinical applicability of LDRT in such a young population. Substantial long-term disability rates in this relatively mild cohort also warrant caution [26]. These findings therefore generate a hypothesis regarding earlier OA and orthovoltage treatment but do not establish treatment-specific efficacy.

## 4 From evidence gaps to a definitive OA evaluation and implementation pathway

The current evidence leaves the role of LDRT in the clinical management of patients with OA unresolved. Uncertainty exists at several successive levels of the translational chain: the OA-specificity of proposed mechanisms, demonstration of biological target engagement in humans, treatment-specific efficacy beyond sham, and identification of responsive phenotypes. Negative sham-controlled trials in unselected, established OA argue against routine use, whereas emerging positive signals justify further hypothesis-driven testing rather than clinical adoption. At present, LDRT cannot be considered a standard OA treatment despite its inclusion in German DEGRO recommendations [16]. Recent multidisciplinary appropriate use criteria may help structure discussion by addressing patient selection, safety assessment, dose, and technique [17], but they do not replace OA-standard sham-controlled trials needed to establish treatment-specific efficacy, responsive phenotypes, and long-term benefit–risk balance. This need has also been emphasized, particularly given the historical reliance on uncontrolled clinical experience in a disease characterized by substantial placebo responses [26].

Addressing these uncertainties requires progress across several interconnected dimensions, spanning patient selection and biological target engagement, radiation delivery and trial methodology, long-term safety and positioning within OA care, and patient, referrer, and health-system feasibility. Fig. 2 summarizes these requirements as a translational roadmap for the definitive evaluation and potential implementation of LDRT in OA.

**Fig. 2:**
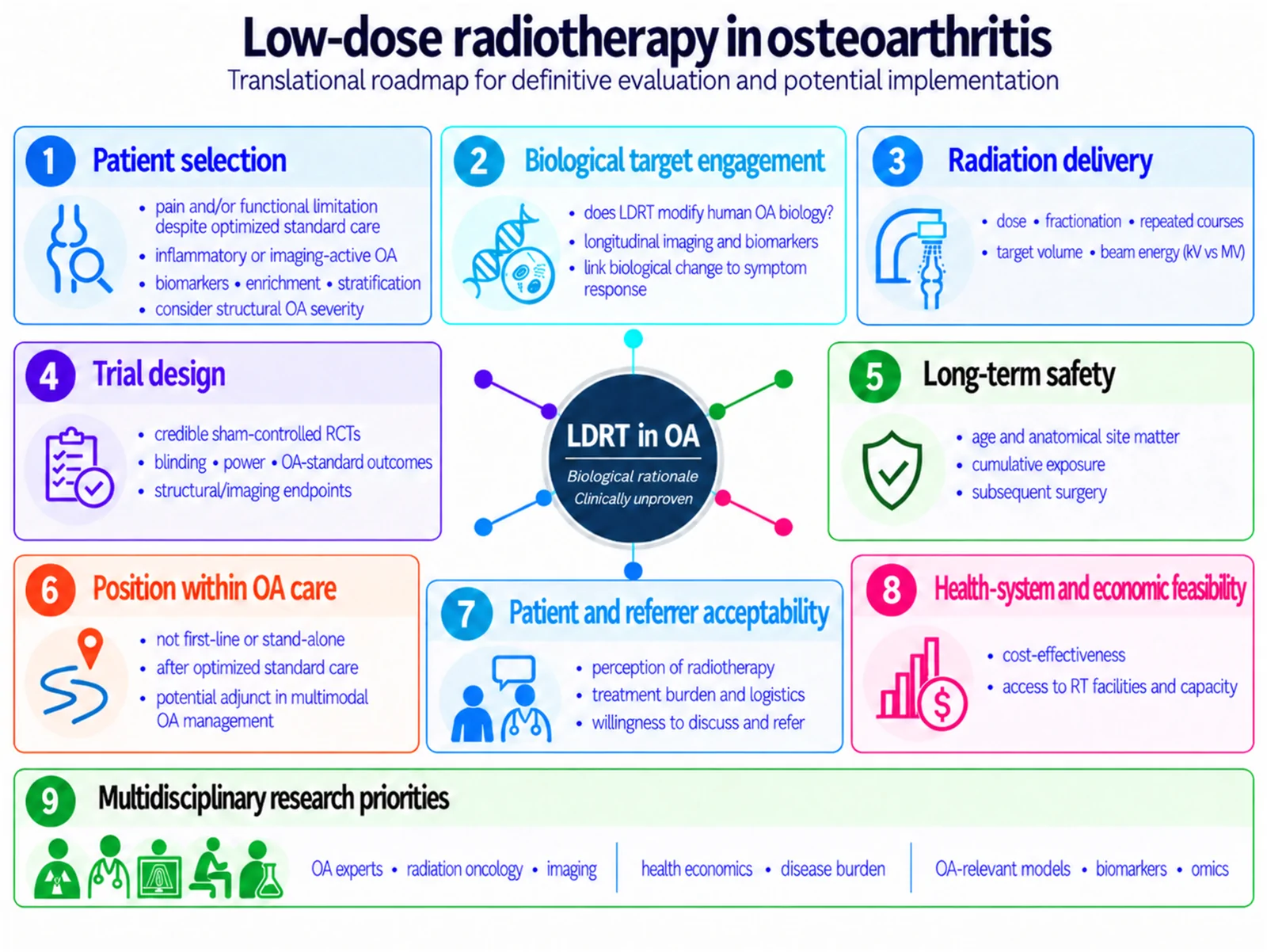
**Translational roadmap for the definitive evaluation and potential implementation of LDRT in osteoarthritis.** This figure summarizes the main requirements identified in Section 4 for the definitive evaluation of low-dose radiotherapy (LDRT) in patients with osteoarthritis (OA) and, if efficacy is demonstrated, for its potential implementation in OA care. At the center, LDRT is presented as having a biological rationale but remaining clinically unproven. The surrounding domains highlight the main unresolved questions across the translational chain [1]: patient selection, including clinically meaningful symptoms despite optimized standard care, consideration of earlier disease stages rather than advanced structural OA, and possible enrichment for inflammatory or imaging-active OA [2]; biological target engagement, that is, whether irradiation modifies relevant human OA pathways [3]; radiation delivery, including dose, fractionation, beam energy, target volume and repeated courses [4]; trial design, requiring credible sham-controlled randomized trials with OA-standard outcomes [5]; long-term safety, according to age, anatomical site, cumulative exposure and subsequent surgery [6]; positioning within OA care, as a possible adjunct after optimized standard management rather than a first-line or stand-alone treatment [7]; patient and referrer acceptability [8]; health-system and economic feasibility; and [9] multidisciplinary research priorities. *The graphical development of this conceptual figure was assisted by ChatGPT (OpenAI), based on scientific content and instructions provided by the authors. The final figure was reviewed and validated by the authors*.

### 4.1 Patient selection: who should actually be studied?

Patient selection is among the most important unresolved issues. Future trials should first enroll patients with clinically meaningful pain and/or functional impairment despite optimized standard OA care.

Because the strongest biological rationale for LDRT is anti-inflammatory and immunomodulatory, prospective enrichment for inflammatory or imaging-active OA is a rational hypothesis to test. Candidate features include clinical inflammatory manifestations or flares, ultrasound-defined synovitis or power Doppler activity, MRI-defined synovitis or effusion, and selected circulating, synovial or tissue-remodeling biomarkers [6], [87]. No biomarker or responder phenotype has yet been validated for LDRT in OA. Nevertheless, exploratory observations from other OA trials suggest that baseline inflammatory features may modify treatment response [88], [89], supporting prospective evaluation of inflammatory enrichment in LDRT trials. Future trials should therefore require clinically meaningful pain and functional impairment, consider earlier disease stages rather than advanced structural OA, and prespecify objective inflammatory features for enrichment and stratification.

### 4.2 Biological target engagement: does irradiation actually modify human OA?

The same imaging and biological measures used for baseline phenotyping can also be repeated after treatment to determine whether LDRT engages the proposed biological target. Longitudinal changes in synovitis, power Doppler activity, MRI inflammatory features or selected biomarkers should therefore be compared with sham and related to clinical response. Demonstrating such target engagement would help distinguish true biological activity from contextual symptom improvement and strengthen the translational link between preclinical mechanisms and clinical efficacy.

### 4.3 Radiation delivery: dose, fractionation, target volume and technique

A definitive evaluation also requires standardization of the radiation intervention.

Dose, fractionation, treatment timing and the role of repeated courses remain incompletely defined. The recent International Benign Target Volume Group consensus provides three-dimensional target-volume definitions for OA and represents an important framework for harmonizing treatment delivery [90]. However, these volumes remain consensus-based and have not yet been prospectively validated as biological or clinical targets.

The prescribed dose alone does not fully describe how radiation is distributed among individual joint tissues. Beam energy, treatment geometry, and target definition all influence this dose distribution. In particular, kilovoltage (kV) and megavoltage (MV) irradiation differ in their depth-dose characteristics, so that the same nominal dose may expose superficial and deeper joint structures differently. Although a recent systematic review found no clear difference in clinical effectiveness between kV and MV LDRT [91], the available evidence was heterogeneous and largely nonrandomized. Whether their different dose distributions translate into distinct biological effects within OA tissues remains uncertain. Future trials should therefore standardize and report treatment technique and target coverage, rather than dose and fractionation alone.

### 4.4 Trial design: proving benefit beyond sham

Methodologically, the decisive question is whether LDRT can demonstrate a clinically meaningful treatment-specific effect beyond sham. Future trials should therefore use credible sham procedures, adequate statistical power, prespecified OA-standard outcomes and sufficiently long follow-up [66], [67]. This approach is being operationalized, for example, in the Knee Osteoarthritis Radiotherapy Trial (KORT) program, which embeds LDRT within a multidisciplinary pathway including optimization of guideline-based conservative OA care before a planned multicenter sham-controlled phase III trial [92]. Both OMERACT–OARSI responder rates and between-group mean differences in validated continuous pain and function outcomes should be considered, recognizing that OA trials are often powered to detect clinically meaningful between-group mean differences rather than differences in responder rates. Additional outcomes should include rescue-medication use, patient global assessment and, where appropriate, structural and imaging outcomes. The protocol should also prespecify how repeat irradiation is handled, with identical decision criteria and procedures in the sham arm.

### 4.5 Long-term safety and repeated exposure

Safety is particularly important in a nonmalignant condition such as OA. Reported acute and late local toxicities after peripheral-joint LDRT are generally limited, with no major treatment-related events in contemporary OA trials apart from more frequent nail changes after hand irradiation [70], [71]. Broader safety considerations, including age, anatomical site and exposure of radiosensitive tissues, have recently been reviewed in detail [17], [93].

The principal long-term concern remains radiation-induced malignancy. At the doses used for benign disease, the estimated absolute risk is low, particularly in older patients treated at peripheral sites, but is of greater concern with younger age, repeated exposure, larger treatment volumes, more central anatomical sites, and irradiation of active bone marrow [93]. More recently, a retrospective cohort of 4699 patients treated for nonmalignant musculoskeletal disorders reported three in-field solid malignancies (two basal cell carcinomas and one non-small cell lung carcinoma) and a small but statistically significant difference in hematologic malignancy-free survival according to proximity to active bone marrow [94]. These observational data cannot establish causality but reinforce the relevance of anatomical site and marrow exposure when assessing long-term risk.

Repeat irradiation requires additional caution. DEGRO allows a second OA treatment course after approximately 6–12 weeks when response is insufficient (6–8 weeks for small joints) and recommends consideration of cumulative organ-at-risk exposure [16]. To minimize late deterministic toxicity, the guidelines recommend a pragmatic lifetime cumulative dose limit of approximately half the corresponding oncological organ tolerance dose, based on the cumulative absolute maximum dose rather than the sum of prescribed doses. For the skin, this corresponds to approximately 25 Gy. This recommendation does not represent a carcinogenic-risk threshold, and no validated cumulative dose constraints have been established specifically for cartilage, synovium, or subchondral bone. Although subsequent arthroplasty incidence has been reported in one open-label randomized cohort [75], whether prior LDRT affects perioperative complications or prosthesis-related outcomes has not been systematically evaluated and should be prospectively documented.

### 4.6 Where could LDRT fit within contemporary OA care?

Even if efficacy were demonstrated, the clinical value of LDRT would depend on where it is positioned within contemporary OA care. OA is not a therapeutic void: education, exercise therapy, weight management when relevant, and multimodal rehabilitation remain central to evidence-based management, while intra-articular corticosteroids may provide short-term relief during inflammatory flares and other injectable therapies, such as platelet-rich plasma or hyaluronic acid, remain debated because of heterogeneous evidence and uncertain durability. LDRT should therefore not be framed as a first-line or stand-alone intervention, but, if validated, as a potential option for carefully selected patients with persistent symptoms despite optimized standard care.

If ultimately validated, LDRT should therefore be integrated within a multimodal OA pathway rather than developed as a parallel radiation-oncology treatment pathway [92].

### 4.7 Patient and referrer acceptability

Implementation would depend not only on patient acceptance but also on the willingness of non-radiation oncology clinicians to discuss and refer patients for LDRT. Radiotherapy may be perceived primarily as a cancer treatment, but whether this limits acceptance among patients with severe symptomatic OA should be assessed empirically rather than assumed. The trade-off may differ in highly symptomatic phenotypes such as erosive hand OA, in which pain and functional impairment can be substantial and may even exceed those reported by patients with treated rheumatoid arthritis [95]. The treatment burden, practical organization of repeated sessions, and patient expectations should therefore be carefully evaluated in future studies.

Similarly, rheumatologists, rehabilitation physicians, orthopedic surgeons, and general practitioners may have limited familiarity with radiotherapy for nonmalignant disease or concerns regarding radiation risk and the strength of the evidence. These barriers should be assessed empirically rather than assumed.

Future studies should therefore incorporate patient-reported acceptability, treatment burden and expectations, as well as referrer knowledge, willingness to refer and preferred positioning of LDRT within existing treatment algorithms.

### 4.8 Health-system and economic feasibility

Even a clinically effective intervention would need to demonstrate acceptable value and feasibility at health-system level. OA is highly prevalent, whereas radiotherapy infrastructure is primarily organized around cancer care. Implementation may also require specific workforce development and education within radiation oncology [96]. Broad implementation could therefore generate substantial opportunity costs, particularly where linear-accelerator capacity is constrained.

Economic evaluation should consider not only the direct cost of simulation, treatment planning and multiple treatment fractions, but also patient travel, time burden, health care utilization, medication use, work productivity and potential effects on downstream interventions such as injections or surgery.

Cost-effectiveness is therefore likely to depend strongly on patient selection, treatment technique, number of fractions, and the durability of any treatment effect.

### 4.9 Translational and multidisciplinary research priorities

These challenges cannot be addressed within a single discipline. Future research should integrate radiation oncology, rheumatology, rehabilitation medicine, orthopedic surgery, pain medicine, imaging, radiobiology, health economics, and musculoskeletal science from trial conception onwards. Mechanistic work should move beyond isolated cell systems toward OA-relevant multicompartment models, including three-dimensional cocultures and organoid-like systems, while clinical programs should integrate biological phenotyping, standardized radiation delivery, target-engagement assessment, and rigorous sham-controlled evaluation. Omics and advanced imaging may further help identify biomarkers and responder profiles and distinguish symptomatic from structural effects.

## 5 Conclusions

Current evidence provides a biological rationale for LDRT in OA but does not support its routine clinical use in patients. The key question is now whether treatment-specific efficacy can be demonstrated in biologically and clinically characterized populations using adequately powered sham-controlled trials that integrate target engagement, standardized radiation delivery, and long-term safety. Resolving this question will require genuine collaboration between OA and radiation experts. LDRT should therefore neither be adopted on precedent nor dismissed on principle, but subjected to the evidentiary standards expected of any contemporary OA intervention.

## Authorship

GC, JSe and OM conceived the overall design, scope and structure of the review. GC, JSe and OM performed the literature appraisal and drafted the initial manuscript. JFK, PD, FL, PB and CR contributed to the interpretation of the literature, critically revised the manuscript for important intellectual content, and provided substantive comments and revisions. All authors contributed to the final interpretation of the evidence, approved the version to be submitted, and confirm that neither this manuscript nor one with substantially similar content has been submitted, accepted or published elsewhere, except as an abstract. GC, JSe and OM take responsibility for the integrity of the work as a whole, from inception to finished article.

## AI use disclosure

During the preparation and revision of this manuscript, the authors used ChatGPT (OpenAI) to assist with translation from French into English, linguistic editing, and the integration of author-directed revisions into the English manuscript. In particular, scientific revisions developed and discussed by the authors, including passages initially revised in French in response to peer-review comments, were translated and integrated with AI assistance to improve clarity, consistency, and readability while preserving the intended scientific meaning.

ChatGPT was also used to assist with the graphical development and refinement of Fig. 1, Fig. 2, based on scientific concepts and instructions provided by the authors.

All scientific interpretations, critical appraisal of the literature, and final editorial decisions remained under the responsibility of the authors. All AI-assisted content was reviewed and edited by the authors, who take full responsibility for the accuracy and integrity of the final manuscript.

## Conflict of interest

JSe reports honoraria from AbbVie, AlfaSigma, Axomove, Fresenius Kabi, Guerbet, IBSA Pharma, Johnson & Johnson, Eli Lilly, Nordic Pharma, Novo Nordisk, Novartis and UCB, outside the submitted work. CR reports honoraria from AbbVie, AlfaSigma, Celltrion, EG Pharma, Johnson & Johnson, Eli Lilly, Novartis, Pfizer and UCB, outside the submitted work. OM reports honoraria from AbbVie, AlfaSigma, Celltrion, EG Pharma, Eli Lilly and UCB, outside the submitted work. The remaining authors declare no competing interests.

No author reports financial relationships with radiotherapy equipment manufacturers or commercial providers of low-dose radiotherapy related to the submitted work.
