# Supplementary material for: Low-dose radiotherapy in patients with osteoarthritis: A multidisciplinary roadmap from biological rationale to definitive evaluation
Source: Osteoarthr Cartil Open. 2026 Nov 3;8(4):100892. doi: 10.1016/j.ocarto.2026.100892 (PMC13770673; doi:10.1016/j.ocarto.2026.100892)
Supplement: Supplementary file 1 [file mmc1.docx]

**Supplementary Table 1 |** Preclinical studies of low-dose radiotherapy in osteoarthritis and related inflammatory models

| **Experimental system** | **Disease / inflammatory context** | **Irradiation schedule** | **Biological compartment / readouts** | **Main preclinical findings** | **Translational interpretation** | **Study** |
| --- | --- | --- | --- | --- | --- | --- |
| **In vitro and ex vivo models** | | | | | | |
| ***Vascular interface and leukocyte trafficking*** | | | | | | |
| In vitro endothelial–leukocyte adhesion assay using a murine endothelial cell line and PBMCs from healthy human donors | IL-1β-activated endothelium | Single-dose X-irradiation, 0.3–3 Gy; IL-1β stimulation after irradiation | Endothelium–leukocyte interface; TGF-β1, IL-6, E-selectin and PBMC adhesion | Reduced PBMC adhesion at 0.3–0.7 Gy; maximal TGF-β1/IL-6 induction at 0.5 Gy; partial reversal by TGF-β1 blockade | Identifies a low-dose anti-adhesive effect potentially relevant to inflammatory cell recruitment | Rödel et al., 2002 (35) |
| In vitro human endothelial cells | TNF-α-activated endothelium | Single-dose X-irradiation, 0.01–2 Gy; TNF-α added 2 h before irradiation | Endothelium; secretion and mRNA expression of IL-8, G-CSF and PDGF-BB | LDRT alone increased mediator release in resting endothelium, whereas it induced an early, dose-selective reduction in TNF-α-induced IL-8 | Shows context- and time-dependent endothelial immunomodulation by LDRT | Schröder et al., 2019 (36) |
| In vitro human endothelial cell–neutrophil co-culture | TNF-α-activated endothelium; direct PMN–endothelium contact | Endothelial cells irradiated before PMN co-culture/TNF-α activation: single 0.3–3 Gy or two fractions 24 h apart | Endothelium–neutrophil interface; CCL20, TNF-α, TGF-β1 and PMN adhesion | TGF-β1-dependent reduction in CCL20 at 0.5–1 Gy, with a minimum at 0.8 Gy; PMN adhesion reduced most at 0.5 Gy | LDRT may limit neutrophil transendothelial migration across activated endothelium | Rödel et al., 2008 (37) |
| In vivo mouse model with abdominal irradiation and intravital assessment of intestinal venules | Acute LPS-induced inflammation | Single 0.3 Gy; LPS challenge before readout; assessment 5–72 h after irradiation | Intestinal venules; leukocyte rolling/adhesion; circulating and intestinal TGF-β1 | Leukocyte adhesion decreased at 5–48 h but not at 72 h; TGF-β1 increased at 5–24 h | Suggests a transient anti-adhesive effect that may inform fraction spacing | Arenas et al., 2008 (39) |
| In vitro human endothelial cell and PBMC models | TNF-α-activated endothelium | Single 0.3–3 Gy; TNF-α before irradiation for redox analyses and after irradiation for adhesion assays | Endothelial redox pathways; GPx, SOD1, Nrf2, ROS and PBMC adhesion | GPx/SOD1/Nrf2 minima at 0.5 Gy, with partial rebound at 0.7–1 Gy; reduction in PBMC adhesion blunted by NAC | Suggests redox-dependent control of the low-dose window for leukocyte adhesion | Large et al., 2015 (38) |
| ***Immune plasticity*** | | | | | | |
| In vitro human THP-1-derived macrophages | LPS-primed, MSU-triggered inflammation | LPS priming before irradiation; single 0.1–1 Gy; MSU 30 min after irradiation | Macrophage inflammatory signaling; IL-1β, p65/NF-κB, p38, Akt and LDH | IL-1β reduced most at 0.5–0.7 Gy, with no significant reduction at 1 Gy; NF-κB signaling reduced | Supports nonlinear dose-dependent macrophage immunomodulation | Lödermann et al., 2012 (40) |
| Ex vivo peritoneal macrophages from BALB/c and C57BL/6 mice | LPS-primed, MSU-triggered inflammation | LPS 16 h before irradiation; single 0.01–1 Gy; MSU 30 min after irradiation | Primary macrophage cytokine output; IL-1β and TNF-α | IL-1β decreased only in BALB/c macrophages at 0.5–0.7 Gy; no effect in C57BL/6 macrophages | Suggests that host radiosensitivity may condition the macrophage response | Frischholz et al., 2013 (41) |
| Ex vivo mouse peritoneal macrophages | LPS-activated macrophages | LPS 16 h before irradiation; single 0.01–2 Gy | Macrophage migration, chemotaxis, cytokines, NF-κB p65, viability and phagocytosis | Transmigration reduced at 0.1–0.5 Gy; IL-1β reduced from 0.5 Gy; phagocytosis preserved | Supports functional macrophage reprogramming without loss of phagocytosis | Wunderlich et al., 2015 (42) |
| In vitro human PBMCs | Lymphocyte apoptosis model | Single 0.1–3 Gy | PBMC apoptosis; CD4+, CD8+ and CD19+ lymphocyte subsets | Discontinuous apoptotic response, with a plateau/inflexion at 0.3–0.7 Gy in most donors and subsets | Supports dose-window-dependent lymphocyte immunomodulation | Kern et al., 1999 (43) |
| In vitro isolated human peripheral immune cells | Immune radiosensitivity model (apoptosis and necrosis) | Single 0.01–60 Gy | Peripheral immune subsets: T cells, B cells, NK cells and monocytes | Monocytes were radioresistant; B-cell and NK-cell viability decreased from 0.3–0.7 Gy, with NK cells most sensitive | Supports subset-specific lymphocyte radiosensitivity | Falcke et al., 2018 (44) |
| Ex vivo PBMCs and FLS from patients with RA | Human RA | PBMCs: 0.5 Gy; FLS: 0.5 or 2 Gy | Apoptosis of CD4+ T cells, CD19+ B cells and RA-derived FLS | CD4+/CD19+ apoptosis increased at 0.5 Gy; FLS apoptosis increased at 2 Gy but not at 0.5 Gy | Supports differential radiosensitivity of lymphoid and stromal compartments | Kim et al., 2024 (45) |
| ***Joint compartments and tissue crosstalk*** | | | | | | |
| In vitro FLS derived from hTNF-α transgenic mice | TNF-driven inflammatory arthritis | Single 0.1–2 Gy | Synovial fibroblast phenotype and apoptosis; cytokines | FLS apoptosis increased from 0.5 Gy; IL-6 decreased at 0.5 Gy; hTNF-α and CXCL1 decreased at 1–2 Gy | Supports dose-specific stromal modulation in inflamed synovium | Deloch et al., 2018 (46) |
| In vitro hTNF-α transgenic mouse macrophages exposed to FLS-conditioned supernatants | TNF-driven inflammatory arthritis; synovial crosstalk | FLS irradiated at 0.1–2 Gy, or macrophages irradiated after exposure to supernatant from non-irradiated FLS | Soluble FLS–macrophage crosstalk; CD206, CD80, CD86 and MHCII | Supernatant from irradiated FLS reduced CD206 expression from 0.5 Gy; irradiated macrophages exposed to supernatant from nonirradiated FLS increased CD206 only at 0.5 Gy | Shows microenvironment-dependent macrophage modulation | Deloch et al., 2019 (47) |
| Human OA chondrocytes and synoviocytes | IL-1β-stimulated, patient-derived OA joint cells | IL-1β 24 h before irradiation; 0.25–2 Gy in one or two fractions | Chondrocytes and synoviocytes; catabolic, mitochondrial and secretory responses | MMP-13 decreased at approximately 0.5–1 Gy; COL2 increased; inverted U-shaped response for mitochondrial β-oxidation/UPR pathway activation; GDF15 increased | OA-specific human-cell model supporting anti-catabolic and stress-response modulation | Kim et al., 2022 (48) |
| Ex vivo human monocyte-derived osteoclasts cultured on bone slices | Healthy-donor monocytes cultured with M-CSF/RANKL | Osteoclast precursors irradiated after attachment; single 0.5–10 Gy or fractionated 0.5–1 Gy | Osteoclast differentiation, fusion, resorbed area, CTX release and NFATc1 translocation | Resorbed area decreased >3-fold at 0.5 Gy, with reduced CTX release; impaired fusion/NFATc1 translocation occurred mainly at ≥2 Gy or after fractionation | Supports modulation of osteoclast function | Eckert et al., 2022 (50) |
| Ex vivo FLS, osteoclasts, osteoblasts and bone marrow from healthy mice | Healthy, noninflamed joint-cell model | Single 0.1–2 Gy | FLS function, osteoclast resorption, osteoblast mineralisation and bone-marrow immune subsets | Minimal changes in FLS; osteoclast pit area decreased from 0.5 Gy; osteoblast mineralization increased at 0.1–0.5 Gy | Supports limited basal toxicity; effects appear context- and inflammation-dependent | Deloch et al., 2018 (51) |
| **In vivo models (non-OA-specific)** | | | | | | |
| In vivo female Lewis rat model with local hind-paw irradiation | Adjuvant-induced arthritis | Local hind-paw irradiation: 5 × 0.5 Gy or 5 × 1 Gy | Paw swelling, ESR, macrophage infiltration, cartilage/bone damage, iNOS and HO-1 | Clinical inflammation and structural damage decreased; iNOS decreased and HO-1 increased; 5 × 0.5 Gy was similar to 5 × 1 Gy | Supports local anti-inflammatory and structure-preserving tissue effects | Hildebrandt et al., 2003 (52) |
| In vivo CIA and K/BxN mouse models with lower-body irradiation | Autoimmune inflammatory arthritis | 0.5 Gy per fraction; CIA: 8 × 0.5 Gy; K/BxN: 4 × 0.5 Gy | Clinical arthritis; dLN immune cells; CD4+/B220+ apoptosis; K/BxN joint structure | Clinical severity decreased; CD4+ and B220+ apoptosis increased in dLN; structural damage decreased in K/BxN joints | Supports disease attenuation (associated with lymphocyte apoptosis) | Kim et al., 2024 (45) |
| In vivo CIA mouse model with whole-body irradiation | Collagen-induced autoimmune arthritis | 0.5 Gy weekly × 5; first dose 3 days before CII/CFA immunisation | Clinical arthritis, bone degradation, splenic cytokines, anti-CII antibodies and Tregs | Incidence, severity and bone degradation decreased; TNF-α, IFN-γ, IL-6 and anti-CII antibodies decreased; CD4+CD25+Foxp3+ Tregs increased | Supports systemic immunoregulation; primarily preventive/peri-induction design | Nakatsukasa et al., 2008 (53) |
| In vivo rat model with whole-body irradiation | FCA-induced adjuvant arthritis | Single 0.5 Gy on day 12, or 2 × 0.25 Gy on days 6 and 12 after FCA | Clinical arthritis, histology, cytokines, RANKL/OPG, NF-κB p65 and MMP-13 | The 2 × 0.25 Gy regimen produced a stronger clinical effect; IL-1β, TNF-α, IL-6, IL-17A and RANKL decreased; IL-10 and OPG increased | Supports anti-inflammatory and anti-erosive signaling | Abdel-Rafei et al., 2022 (54) |
| K/BxN serum-transfer mouse model with unilateral hind-limb irradiation | Inflammatory arthritis/pain model | Local 0.5 Gy on day 3 after serum transfer; contralateral limb shielded | Bone marrow, blood, serum cytokines and histology | Bone marrow T cells decreased bilaterally; CD8+/CD4+ balance shifted towards CD4+ cells; dendritic cells decreased in the irradiated limb; IL-4 and IL-6 increased, whereas IL-17A decreased | Supports systemic immune effects after focal LDRT | Weissmann et al., 2022 (55) |
| In vivo hTNF-α transgenic mouse model with local hind-limb irradiation | TNF-driven inflammatory arthritis | Single local 0.5 Gy | Grip strength, synovial inflammation, bone erosion, osteoclasts and neutrophils | Inflammatory and erosive areas decreased; osteoclasts and neutrophils decreased; early grip strength was preserved | Supports local modulation of bone–inflammation interactions | Deloch et al., 2018 (46) |
| **In vivo models (OA-specific)** | | | | | | |
| In vivo rat model | Post-traumatic/surgical OA induced by ACLT or DMM knee injury | Irradiation 3 weeks after surgery; 5 × 0.5 Gy or 5 × 1 Gy | Cartilage histology, OARSI score and TRAP-positive osteoclast activity | OARSI score decreased from 3.7 (sham) to 2.8 and 1.8 with 5 × 0.5 Gy and 5 × 1 Gy, respectively; TRAP-positive osteoclast clusters decreased | Provides OA-specific in vivo evidence and a structural signal | Kim et al., 2022 (48) |
| In vivo rat model | Chemically induced OA-like inflammatory pain model | Irradiation 1 week after MIA; 5 × 0.5 Gy or 1 × 1 Gy | Cartilage, synovium, macrophages, behavior and spinal cord | Functional and analgesic signal; cartilage damage and synovitis decreased; spinal IFN-β1, TNF-α, IL-1β, CCL2 and CXCL1 decreased; GFAP decreased | Supports peripheral and spinal anti-inflammatory pain modulation | Ma et al., 2025 (57) |

**Abbreviations:** ACLT, anterior cruciate ligament transection; Akt, protein kinase B; BM, bone marrow; CCL, C-C motif chemokine ligand; CFA, complete Freund adjuvant; CIA, collagen-induced arthritis; CII, type II collagen; COL2, type II collagen; CTX, C-terminal telopeptide of type I collagen; DC, dendritic cell; dLN, draining lymph node; DMM, destabilization of the medial meniscus; ESR, erythrocyte sedimentation rate; FCA, Freund complete adjuvant; FLS, fibroblast-like synoviocyte; G-CSF, granulocyte colony-stimulating factor; GDF15, growth differentiation factor 15; GFAP, glial fibrillary acidic protein; GPx, glutathione peroxidase; HO-1, heme oxygenase 1; IFN, interferon; IL, interleukin; iNOS, inducible nitric oxide synthase; LDRT, low-dose radiotherapy; LDH, lactate dehydrogenase; LPS, lipopolysaccharide; M-CSF, macrophage colony-stimulating factor; MHCII, major histocompatibility complex class II; MIA, monosodium iodoacetate; MMP, matrix metalloproteinase; MSU, monosodium urate; NAC, N-acetylcysteine; NF-κB, nuclear factor-κB; NFATc1, nuclear factor of activated T cells 1; NK, natural killer; Nrf2, nuclear factor erythroid 2-related factor 2; OA, osteoarthritis; OARSI, Osteoarthritis Research Society International; OPG, osteoprotegerin; PBMC, peripheral blood mononuclear cell; PDGF-BB, platelet-derived growth factor-BB; PMN, polymorphonuclear neutrophil; RA, rheumatoid arthritis; RANKL, receptor activator of NF-κB ligand; ROS, reactive oxygen species; RT, radiotherapy; SOD1, superoxide dismutase 1; TGF-β1, transforming growth factor-β1; TNF-α, tumor necrosis factor-α; TRAP, tartrate-resistant acid phosphatase; Treg, regulatory T cell; UPR, unfolded protein response.
